# Instantaneous Real-Time Kinematic Decimeter-Level Positioning with BeiDou Triple-Frequency Signals over Medium Baselines

**DOI:** 10.3390/s16010001

**Published:** 2015-12-22

**Authors:** Xiyang He, Xiaohong Zhang, Long Tang, Wanke Liu

**Affiliations:** 1 School of Geodesy and Geomatics, Wuhan University, 129 Luoyu Road, Wuhan 430079, China; hexiyang704814@126.com (X.H.); wkliu@sgg.whu.edu.cn (W.L.); 2 Collaborative Innovation Center for Geospatial Technology, 129 Luoyu Road, Wuhan 430079, China

**Keywords:** BeiDou, triple frequency, carrier-smoothed code (CSC), extra-wide-lane, wide-lane, ambiguity resolution, differential positioning

## Abstract

Many applications, such as marine navigation, land vehicles location, *etc*., require real time precise positioning under medium or long baseline conditions. In this contribution, we develop a model of real-time kinematic decimeter-level positioning with BeiDou Navigation Satellite System (BDS) triple-frequency signals over medium distances. The ambiguities of two extra-wide-lane (EWL) combinations are fixed first, and then a wide lane (WL) combination is reformed based on the two EWL combinations for positioning. Theoretical analysis and empirical analysis is given of the ambiguity fixing rate and the positioning accuracy of the presented method. The results indicate that the ambiguity fixing rate can be up to more than 98% when using BDS medium baseline observations, which is much higher than that of dual-frequency Hatch-Melbourne-Wübbena (HMW) method. As for positioning accuracy, decimeter level accuracy can be achieved with this method, which is comparable to that of carrier-smoothed code differential positioning method. Signal interruption simulation experiment indicates that the proposed method can realize fast high-precision positioning whereas the carrier-smoothed code differential positioning method needs several hundreds of seconds for obtaining high precision results. We can conclude that a relatively high accuracy and high fixing rate can be achieved for triple-frequency WL method with single-epoch observations, displaying significant advantage comparing to traditional carrier-smoothed code differential positioning method.

## 1. Introduction

Over the past decades, different GNSS techniques have been developed by researchers, which have made remarkable contributions to scientific applications (e.g., geodesy, remote sensing, space and fundamental physics) [1,2] and engineering services (e.g., surveying, navigation, and timing) [3,4]. With regard to the navigation and positioning techniques of GNSS, they can be divided into two main categories: absolute positioning and relative positioning. Absolute positioning mainly includes standard point positioning (SPP) and precise point positioning (PPP). Relative positioning mainly includes differential DGNSS (DGNSS) and real-time kinematic (RTK) positioning techniques. Different advantages as well as disadvantages are shown for these techniques.

SPP, as the simplest positioning technique, can realize real-time positioning using a single receiver with broadcast ephemeris. Its applications are mainly in navigation. The main disadvantage of SPP is that the accuracy is limited to several to tens meters [5]. Using the PPP technique, positioning with a single receiver can also be implemented, but providing higher centimeter level accuracy [6,7]. However, precise ephemeris and clock products must be provided for PPP. In addition, a convergence time of about thirty minutes is required to reach high precision results [8].

The real-time kinematic (RTK) positioning technique, which was first investigated by Kleusberg [9], Cannon [10] and Schwarz *et al.* [11], implements positioning using two or more receivers. The rover position is determined by forming double-differenced observations with the reference station. Using the RTK technique, one can realize fast precise centimeter level positioning [12]. However, the applications of RTK are limited to short distances (e.g., <10 km). For medium and long distances (e.g., >10 km), a certain time duration (usually 10–40 min for GPS) is needed first for fixing the ambiguities due to the existence of residual atmospheric errors [13].

Using multiple reference stations with inter-receiver distances of several tens of kilometers, the network-RTK [14] and PPP-RTK [15] techniques can be implemented, which are extensions of RTK and PPP, respectively. In this way, the atmospheric errors in a rover station can be modeled or eliminated rapidly, thus improving the positioning performance. For network-RTK, the distance can be extended to several tens of kilometers [14]. For PPP-RTK, high-precision results can be obtained rapidly, e.g., within several seconds [15,16]. The main disadvantage of these two techniques is that they are unavailable in some areas, like marine areas, due to the difficulty of establishing reference stations. PPP-RTK using global-scale networks were also proposed by some researchers [17]. However, at least five to ten minutes are needed for ambiguity fixing for this technique.

Differential GNSS (DGNSS) positioning is a positioning technique where the user's position is determined more accurately by applying differential corrections, derived from a fixed ground-based reference station [18]. DGNSS was invented in the mid-1980s, and was called DGPS at that time. When implementing DGNSS positioning, both original code and carrier-smoothed code (CSC) measurements can be used. The positioning accuracy of original code-based DGNSS is usually at meter level [18]. If one requires decimeter level positioning, CSC measurements usually are needed. The reason is that the impact of noise and multipath code errors can be reduced by applying carrier-smoothing techniques [19,20]. Apparently but unfortunately, a certain time duration (usually several minutes) is also needed for carrier smoothing the code measurements to achieve a relatively high accuracy.

As can be deduced, all these GNSS-based positioning techniques operate under a set of constraints. These constraints include baseline length, attainable accuracy, time-to-solution, instrumentation, operational modes, and so on [21]. Some applications, such as marine navigation, aircraft landing guidance and land vehicles location, *etc*., require obtaining the user positions in a rather short time with certain positioning accuracy (decimeter-level) and certain coverage (e.g., <100 km). In some areas like marine areas, it is also hard to establish multiple-reference stations in the surrounding areas. For such applications, CSC-based DGNSS is currently the most suitable method, since it can obtain the user positions in a relatively short time with decimeter level accuracy using just a single or a few reference stations. However, this technique still suffers some time cost for code carrier smoothing, which has been described above.

Currently, with the two new and emerging BeiDou and Galileo constellations as well as the ongoing modernization of GPS and GLONASS, more multi-frequency signals will become available for most users [22]. As to the current BDS constellation, all BDS satellites can transmit triple-frequency signals to the users. With triple-frequency signals, more useful combinations can be formed, which can improve the performance of ambiguity resolution (AR). Significant research efforts have been made in the past decade to make use of the multi-frequency signals for improving AR (ambiguity resolution). Harris [23] and Forssell *et al*. [24] proposed the Three Carrier Ambiguity Resolution (TCAR) method. Jung [25] proposed the Cascade Integer Resolution (CIR) method. Feng [26] presented the triple-frequency AR method using geometry-based model. Feng and Li [27] studied the positioning accuracy of wide-lane (WL) signals using GPS measurements. In these studies, only theoretical analysis or simulation test performed. With regard to BDS, some studies [28,29] with real observations are implemented. However, these studies concentrated on the positioning for short baselines or fixing the ambiguities of narrow-lane over medium and long distances, which still requires long observation time.

In this contribution, we aim to develop a new real time differential GNSS positioning technique by using WL measurements obtained from BDS triple-frequency signals. This technique can realize instantaneous real-time kinematic positioning over medium distances with decimeter level precision. The fixing rate and positioning accuracy are investigated in detail. The performance is compared with those of dual-frequency WL case and CSC differential positioning case.

## 2. Wide-Lane Based Differential Positioning

In this section, the models of differential GNSS positioning using WL measurements are introduced. The case with regard to medium distances is focused on here.

### 2.1. Ambiguity Resolution Models

In medium and long baseline (>10 km) cases, residual atmospheric delays will affect the ambiguity resolution, therefore, the atmospheric errors have to be taken into account. There are two methods to deal with the residual atmospheric errors. The first one is to form combinations that can eliminate atmospheric errors or can reduce the impact of atmospheric errors to an extent that can be ignored, e.g., the Hatch-Melbourne-Wübbena (HMW) combination [19,30,31]. The second one is to estimate the residual atmospheric delays as unknown parameters. Here, we focus on the method of forming combinations.

With triple-frequency or multi-frequency signals, more useful combinations can be formed [26,32,33], such as EWL combinations. General triple-frequency phase combinations can be defined as [26]:

(1) $$\Delta \nabla \phi_{\left(i,j,k\right)}=\frac{i\cdot f_{1}\cdot \Delta \nabla \phi_{1}+j\cdot f_{2}\cdot \Delta \nabla \phi_{2}+k\cdot f_{3}\cdot \Delta \nabla \phi_{3}}{i\cdot f_{1}+j\cdot f_{2}+k\cdot f_{3}}$$

where the combination coefficients *i*, *j*, *k* are integers, and *f*_1_, *f*_2_, *f*_3_, are three signal frequencies corresponding to B1, B3 and B2 of BDS or L1, L2 and L5 of GPS, respectively. The DD (double-differenced) phase measurements of three original DD observations in meters are Δφ_1_, Δφ_2_, Δφ_3_, corresponding to, *f*_1_, *f*_2_ and *f*_3_ According to [26], the phase linear combinations can be classified as four categories: extra-wide-lane (EWL, λ ≥ 2.93 m), wide-lane (WL, 0.75 ≤ λ < 2.93 m), medium-lane (ML, 0.19 m ≤ λ < 0.75 m) and narrow-lane (NL, λ < 0.19 m) combinations.

Taking atmospheric errors into account, double-differenced phase and code combinations can be expressed as:

(2) $$\begin{array}{l} \Delta \nabla \phi_{\left(i,j,k\right)}=\Delta \nabla \rho -\beta_{\left(i,j,k\right)}\Delta \nabla I+\Delta \nabla T+\lambda_{\left(i,j,k\right)}N_{\left(i,j,k\right)}+\varepsilon_{\Delta \nabla \phi }\\ \Delta \nabla P_{\left(l,m,n\right)}=\Delta \nabla \rho +\beta_{\left(l,m,n\right)}\Delta \nabla I+\Delta \nabla T+\varepsilon_{\Delta \nabla P} \end{array}$$

where *β* is the ionospheric scale factor, the combination coefficients *l*, *m*, *n* are integers, *ρ* is the geometrical distance, *I* is the ionospheric delay on the first frequency, *T* is the tropospheric delay, *N* is the ambiguity of phase combination, *λ* is the wavelength of phase combination and *ε* is the residual error.

Based on the former studies [26,33], several optimal and useful EWL combinations can be formed, for example, (0, 1, −1) and (1, −5, 4) of BDS. Considering the effects of the phase noise standard deviation and atmospheric errors, total noise level can be defined [26], which is the ratio of the sum of errors and the wavelength. According to [26], the total noise level of these EWL combinations is lower than 0.3 cycles in medium baselines case. Therefore, when implementing AR using these combinations, it is safe to ignore the atmospheric errors.

Theoretically, at least two independent EWL combinations can be formed using triple-frequency signals. The ambiguities of these two independent EWL combinations are determined first. A WL combination then can be derived by combining any of two independent EWL combinations. Therefore, we can fix the ambiguities of two EWL combinations firstly, and then form a WL signals from these two ambiguity-recovered EWL combinations for positioning. Theoretically, implementing wide-lane ambiguity resolution using this method is easier than that in dual-frequency case, because such EWL combinations cannot be found in dual-frequency case.

We will introduce the procedures of this method in detail in the following paragraphs. Two independent EWL combinations (EWL-I and EWL-II) have to be selected first, as listed in Table 1. The characteristics of these selected combinations are also shown. As can be seen, the wavelength of the EWL combinations is up to several meters. Code combinations also have to be formed to constrain the phase combinations. Table 2 shows the combinations we selected.

**Table 1:** Characteristics of the selected phase combinations.

Constellation	Combinations		Wavelength (m)	Ionospheric Scale Factor (m)	Noise Scale Factor (m)
BDS	EWL-I	(0, 1, −1)	4.8842	−1.5915	28.5287
	EWL-II	(1, −5, 4)	6.3707	0.6521	172.6135
GPS	EWL-I	(0, 1, −1)	5.8610	−1.7186	33.24
	EWL-II	(1, −6, 5)	3.2561	−0.0744	103.80

**Table 2:** Characteristics of the selected code combinations.

Constellation	Combinations	Ionospheric Scale Factor (m)	Noise Scale Factor (m)
BDS	(0, 1, 1)	1.5915	28.5287
	(1, 0, 0)	1.0000	1.0
GPS	(0, 1, 1)	1.7186	33.24
	(1, 0, 0)	1.0000	1.0

The first step is to resolve the ambiguity of the first EWL combination $\Delta \nabla \phi_{EWL-I}$. Both geometry-free and geometry based models can be used. The geometry-free model for EWL-I combination is as follows:

(3) $${\overset{⌣}{N}}_{EWL-I}={\left[\frac{\Delta \nabla P_{\left(0,1,1\right)}-\Delta \nabla \phi_{EWI-I}}{\lambda_{EWL-I}}\right]}_{round-off}$$

where *P* indicates the code combinations. *λ* indicates the wavelength $\overset{⌣}{N}$ indicates the fixed ambiguities. The geometry-based model for EWL-I combination can be written as:

(4) $$\left[\begin{array}{c} \Delta \nabla P_{\left(0,1,1\right)}\\ \Delta \nabla \phi_{EWL-I} \end{array}\right]=\left[\begin{array}{cc} A & 0\\ A & E\cdot \lambda_{EWL-I} \end{array}\right]\left[\begin{array}{c} X\\ N_{EWL-I} \end{array}\right]+\left[\begin{array}{c} \varepsilon_{\Delta \nabla P_{\left(0,1,1\right)}}\\ \varepsilon_{\Delta \nabla \phi_{EWL-I}} \end{array}\right]$$

where *E* is the identity matrix. *N* indicates the ambiguities. *X* indicates the baseline estimates, such as position. *A* indicates the design matrix. *ε* is the residual errors. As the ionospheric scale factor of Δ∇*P*_(0,11)_ is equal to that of Δ∇*φ_EWL-Ι_*, the ionspheric error can be completely eliminated. The tropospheric error is also eliminated. When using geometry-free model, rounding method is always used. When using geometry-based model, the Integer Least Squares (ILS) method is always used, which was investigated by many researchers [34,35,36].

The second step is to resolve the ambiguity of the second EWL combination Δ∇*φ_EWL-∏_* according to the following equation. Also, both geometry-free and geometry based models can be used. The geometry-free model is as follows:

(5) $${\overset{⌣}{N}}_{EWL-II}={\left[\frac{\Delta \nabla P_{\left(1,0,0\right)}-\Delta \nabla \phi_{EWL-II}}{\lambda_{EWL-II}}\right]}_{round-off}$$

The geometry-based model can be written as:

(6) $$\left[\begin{array}{c} \Delta \nabla P_{\left(1,0,0\right)}\\ \Delta \nabla \phi_{EWL-II} \end{array}\right]=\left[\begin{array}{cc} A & 0\\ A & E\cdot \lambda_{EWL-II} \end{array}\right]\left[\begin{array}{c} X\\ N_{EWL-II} \end{array}\right]+\left[\begin{array}{c} \varepsilon_{\Delta \nabla P_{\left(1,0,0\right)}}\\ \varepsilon_{\Delta \nabla \phi_{EWL-II}} \end{array}\right]$$

As the ionospheric factor of Δ∇*P*_(0,11)_ is not equal to that of Δ∇*φ_EWL-∏_*, the ionspheric error cannot be completely eliminated. However, the tropospheric error is eliminated.

Based on the above two independent resolved EWL ambiguities, the ambiguity of WL combination Δ∇*φ*_(1,0,–1)_ or Δ∇*φ*_(1,–1,0)_ can be computed according to the linear relationship, e.g., ${\overset{⌣}{N}}_{\left(1,0,-1\right)}=5\cdot {\overset{⌣}{N}}_{\left(0,1,-1\right)}+{\overset{⌣}{N}}_{\left(1,-5,4\right)}$. Then, we can derive the WL observables which can be considered as precise pseudorange observables:

(7) $$\Delta \nabla {\tilde{\Phi }}_{WL}=\Delta \nabla \phi_{WL}+\lambda_{WL}{\overset{⌣}{N}}_{WL}$$

The success rate of WL AR is dependent on the success rate of the two EWL combinations. For dual-frequency signals, HMW combinations can be formed, which eliminate both geometric terms (including clock errors, tropospheric delay, *etc*.) and ionospheric delays (first-order). In the double-difference case, only wide-lane ambiguities, multipath and measurement noise remain. Therefore, the ambiguities of WL measurement can be computed conveniently. Since the wavelength of the WL measurement is longer than 80 cm, we can fix its ambiguity much easier than that of original measurements [17,37].

The formula of DD HMW combination can be expressed as:

(8) $$\Delta \nabla L_{HMW}=\left[\frac{\left(f_{1}\phi_{1}-f_{2}\phi_{2}\right)}{f_{1}-f_{2}}-\frac{\left(f_{1}P_{1}+f_{2}P_{2}\right)}{f_{1}+f_{2}}\right]/\lambda_{WL12}=N_{WL12}+\varepsilon_{HMW}$$

where *f*_1_ and *f*_2_ are the frequencies of the signals; $\varepsilon_{HMW}$ denotes the residual errors and observational noise of HMW combination, which is in units of wide-lane cycles. $\lambda_{WL12}$ is the wavelength of wide-lane measurements, which can be computed as follows:

(9) $$\lambda_{WL12}=\frac{c}{\left(f_{1}-f_{2}\right)}$$

where *c* denotes the speed of light. $N_{WL12}$ is the ambiguity of wide-lane measurements, which is defined as:

(10) $$N_{WL12}=N_{1}-N_{2}$$

where *N*_1_ and *N*_2_ are the ambiguities of original measurements.

Using geometry-free model, WL ambiguities can be obtained:

(11) $${\overset{⌣}{N}}_{WL}={\left[\Delta \nabla {\bar{L}}_{HMW}\right]}_{round-off}$$

where $\Delta \nabla {\bar{L}}_{HMW}$ indicates the averaged value of DD HMW combination. According to former studies [17,37], ten to thirty minutes are usually needed for averaging.

### 2.2. Positioning Model

After the ambiguities of wide-lane observations are fixed, positioning with wide-lane observations can be implemented. The model is as follows:

(12) $$\Delta \nabla {\tilde{\Phi }}_{WL}=AX+\varepsilon_{\Delta \nabla \phi_{WL}}$$

The estimated parameters are only user positions. Since the atmospheric errors are not parameterized or eliminated, this model still suffers from the impact of atmospheric errors. The positioning accuracy are analyzed in detail in the section entitled “Theoretical analysis”.

## 3. Code-based Differential Positioning

The positioning accuracy of the proposed method will be compared with the code-based differential positioning, therefore, the code-based DGNSS model will be briefly introduced here. Code-based DGNSS uses code measurements for positioning. Based on the similarity of the atmospheric errors of base and rover stations, the corrections produced by base station are used for the positioning of rover station.

In base station, corrections at time *t*_0_ are computed as follows:

(13) $$PRC^{S}\left(t_{0}\right)=\tilde{P}-\rho =dt^{s}-dt^{r}+T+I+\varepsilon_{\tilde{P}}$$

where $\tilde{P}$ represents the original code or carrier-smoothed code; *ρ* represents the geometrical distance between satellite and receiver; *dt^s^* represents the satellite clock errors; *dt^r^* represents the receiver clock errors; *T* represents the tropospheric delays; *I* represents the ionospheric delays; $\varepsilon_{\tilde{P}}$ represents the measurement noise.

The positioning model of the rover station is as follows:

(14) $$v=\tilde{P}-\left(dt^{s}-dt^{r}+T+I\right)+\varepsilon_{\tilde{P}}=P-PRC^{S}\left(t\right)+\varepsilon_{\tilde{P}}$$

where *t* indicates the time when a user received the correction for positioning. *PRC*^S^(*t*) is computed according to the following equation:

(15) $$PRC^{S}\left(t\right)=PRC^{S}\left(t_{0}\right)+RRC^{S}\left(t_{0}\right)\left(t-t_{0}\right)$$

When $\tilde{P}$ represents the original code, the model is original code based differential positioning method. When *P* represents the carrier-smoothed code, the model become carrier-smoothed code based differential positioning method. Applying least square method, rover position can be obtained.

For code carrier-smoothing, the general formula is as follows:

(16) $${\bar{P}}_{\left(n\right)}=\phi_{\left(n\right)}+\frac{1}{n}\sum_{i=1}^{n}\left(P_{\left(i\right)}-\phi_{\left(i\right)}\right)$$

where *n* represents the number of epochs. *i* indicates at *i*th epoch. *P* and *φ* can be original measurements or combinations. Its recursive form can be written as:

(17) $$\{\begin{array}{ll} {\bar{P}}_{\left(n\right)}=P_{\left(1\right)} & \text{for }n=1\\ {\bar{P}}_{\left(n\right)}=\frac{1}{n}P_{\left(n\right)}+\frac{n-1}{n}\left({\bar{P}}_{\left(n-1\right)}+\phi_{\left(n\right)}-\phi_{\left(n-1\right)}\right) & \text{otherwise} \end{array}$$

There are several computing methods for code carrier-smoothing. Hatch [19], Goad [20] and McGraw [38] investigated the carrier-smoothing algorithm. McGraw [38] proposed an ionospheric divergence-free carrier-smoothing algorithm, *P* and *φ* adopt the following form:

(18) $$\{\begin{array}{l} P=P_{1}\\ \phi =\frac{f_{1}^{2}+f_{2}^{2}}{f_{1}^{2}-f_{2}^{2}}\phi_{1}-\frac{2f_{2}^{2}}{f_{1}^{2}-f_{2}^{2}}\phi_{2} \end{array}$$

Because the ionospheric scale factor of the carrier phase combination part is equal to 1, and its sign is same as that of code measurement, this algorithm will not be affected by the ionospheric delay divergence.

## 4. Theoretical Analysis

For carrier phase measurements, high positioning accuracy can be achieved only when its ambiguities are fixed. Therefore, the fixing rate of the ambiguities decides the accuracy and reliability of the positioning results in large extent. In this section, the fixing rate of WL AR using dual-frequency signals and triple-frequency signals are analyzed theoretically. The wide-lane observational errors are larger than those of narrow-lane or original carrier phase measurements, thus the positioning accuracy is lower than that of narrow-lane measurements. It is necessary to figure out what positioning accuracy the wide-lane signals can achieve over medium distances. Therefore, the positioning accuracy is also evaluated theoretically, and compared with that of carrier-smoothed code differential positioning in this section.

For convenience, we define:

(a) DFWL-DGNSS: WL differential positioning with dual-frequency signals;
(b) TFWL-DGNSS: WL differential positioning with triple-frequency signals;
(c) ORC-DGNSS: original-code based differential positioning;
(d) CSC-DGNSS: carrier-smoothed-code based differential positioning.

### 4.1. Theoretical Fixing Rate

According to [39], when using rounding method, the theoretical fixing rate can be expressed as:

(19) $$P_{succ}=\int_{-\infty }^{\frac{0.5-\mu_{\hat{N}}}{\sigma_{\hat{N}}}}\frac{1}{\sqrt{2\pi }}e^{-z^{2}/2}dz-\int_{-\infty }^{\frac{-0.5-\mu_{\hat{N}}}{\sigma_{\hat{N}}}}\frac{1}{\sqrt{2\pi }}e^{-z^{2}/2}dz$$

where $\sigma_{\hat{N}}$ is the standard deviation of the errors of the float ambiguities, $\mu_{\hat{N}}$ is the bias of the float ambiguities. We use this formula to assess the theoretical fixing rate of TFWL-DGNSS and DFWL-DGNSS. Since Equation (19) corresponds to a rounding method, some difference will be caused to the fixing rate when TFWL-DGNSS uses ILS method. However, the fixing rate of ILS method used in geometry-based model will be higher or at least equivalent to that of rounding method used in geometry-free model [40]. In addition, DFWL-DGNSS uses the rounding method. Therefore, it is still reasonable for us to evaluate the fixing rate using Equation (19).

From the analysis of Section 2.1, we can learn that the float ambiguities of the EWL-I combination are not affected by the atmospheric errors, whereas the float ambiguities of the EWL-II combination are affected by the residual atmospheric errors.

Assuming that the standard errors of undifferenced phase and code measurements are $\sigma_{P_{\text{u}}}$ = 0.3 m and $\sigma_{\phi_{u}}$ = 0.003 m, respectively and taking the double-differenced (DD) case into account, the standard errors of the float ambiguities for BDS WL (computed based on the HMW combination), EWL-I and EWL-II combinations will be $\sigma_{{\hat{N}}_{WL}}$ = 0.565 cycles, $\sigma_{{\hat{N}}_{EWL-I}}$ =0.087 cycles and $\sigma_{{\hat{N}}_{EWL-II}}$ = 0.188 cycles, respectively. The values are computed based on Equations (3), (5) and (8) according to the error propagation law. Since the float ambiguities of EWL-II are affected by the atmospheric errors, biases might exist in the float ambiguities. Assuming that residual ionospheric delay is 0.40 m, the caused biases for EWL-II will not exceed 0.11 cycles. We roughly assume that the residual atmospheric errors cause a bias of 0.11 cycles to the EWL-II float ambiguities. Therefore, the forms of the distribution for the float ambiguities of WL, EWL-I and EWL-II are $\varepsilon_{{\hat{N}}_{WL}}∼N\left(0,0.565\right)$, $\varepsilon_{{\hat{N}}_{EWL-I}}∼N\left(0,0.087\right)$ and $\varepsilon_{{\hat{N}}_{EWL-II}}∼N\left(0.11,0.188\right)$, respectively. In a similar way, the distributions of the float ambiguities for GPS WL, EWL-I and EWL-II combinations can be obtained.

Based on Equation (19), we computed the theoretical fixing rate of WL and two EWL combinations, as shown in Table 3. As can be seen, the fixing rates of the BDS EWL-I and EWL-II combinations are higher than 98.0%, whereas the fixing rate of the BDS WL combination is lower than 65%. The values indicate that the ambiguities of the selected two EWL combinations can be resolved with a rather high fixing rate.

**Table 3:** Theoretical fixing rate of WL and EWL combinations.

Combinations	Theoretical Fixing Rate	
	BDS	GPS
WL	62.38%	62.65%
EWL-I	~100.00%	~100.00%
EWL-II	98.04%	91.67%

### 4.2. Theoretical Accuracy

We first analyze the accuracy of the CSC measurements. Equation (16) can be rewritten as:

(20) $${\bar{P}}_{\left(n\right)}=\frac{n-1}{n}\phi_{\left(n\right)}-\frac{1}{n}\sum_{i=1}^{n-1}\phi_{\left(i\right)}+\frac{1}{n}\sum_{i=1}^{n}P_{\left(i\right)}$$

Assuming that the accuracy of DD code measurement is $\sigma_{P}$, the accuracy of DD carrier phase measurement is $\sigma_{\phi_{c}}$, then the accuracy of the carrier-smoothed code measurements can be computed as follows:

(21) $$\sigma_{\bar{P}}=\sqrt{\frac{n-1}{n}\sigma_{\phi_{c}}^{2}+\frac{1}{n}\sigma_{P}^{2}}$$

where:

(22) $$\sigma_{\phi_{c}}=\sqrt{{\left(\frac{f_{1}^{2}+f_{2}^{2}}{f_{1}^{2}-f_{2}^{2}}\right)}^{2}+{\left(\frac{2f_{2}^{2}}{f_{1}^{2}-f_{2}^{2}}\right)}^{2}}\sigma_{\phi }=6.24\sigma_{\phi }$$

where $\sigma_{\phi }$ indicates the accuracy of original DD carrier phase measurement. Then, we have:

(23) $$\sigma_{\bar{P}}=\sqrt{\frac{n-1}{n}{\left(6.24\sigma_{\phi }\right)}^{2}+\frac{1}{n}\sigma_{P}^{2}}$$

For wide lane measurements, only carrier phase measurements have to be taking into account, the accuracy can be computed as follows:

(24) $$\sigma_{\phi_{WL}}=\sqrt{{\left(\frac{f_{1}}{f_{1}-f_{2}}\right)}^{2}\sigma_{\phi_{1}}^{2}+{\left(\frac{f_{2}}{f_{1}-f_{2}}\right)}^{2}\sigma_{\phi_{2}}^{2}}=\sqrt{{\left(\frac{f_{1}}{f_{1}-f_{2}}\right)}^{2}+{\left(\frac{f_{2}}{f_{1}-f_{2}}\right)}^{2}}\sigma_{\phi }=6.87\sigma_{\phi }$$

**Figure 1:**
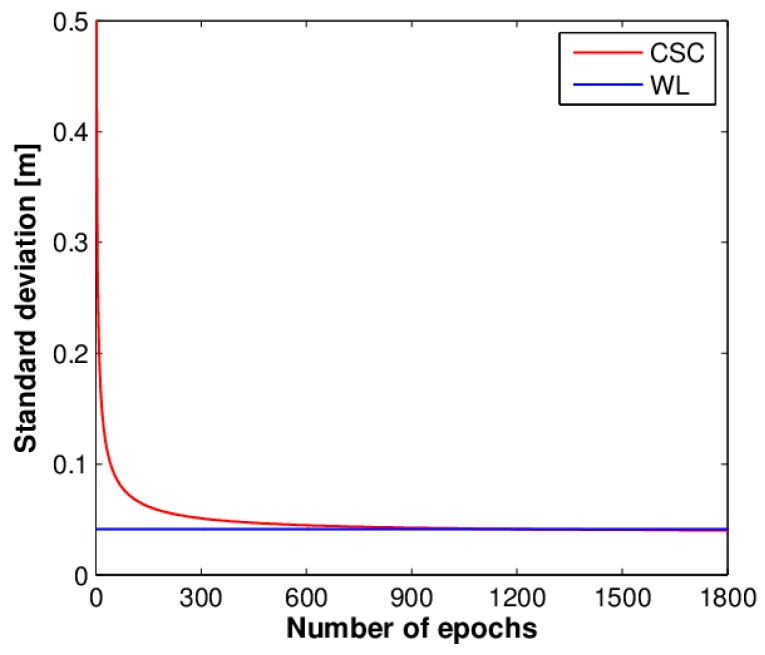
Standard deviation of CSC measurements and WL measurements *versu*s the number of epochs.

Assuming that $\sigma_{P_{u}}$ = 0.3 m and $\sigma_{\phi_{u}}$ = 0.003 m and taking the double-differenced (DD) case into account, we calculated the standard deviation of CSC measurements and WL measurements *versus* the number of epochs, as shown in Figure 1. From the above two equations and Figure 1, we can learn that the accuracy of the WL measurement is comparable to that of the CSC measurement when a sufficient number of epochs are used. However, the accuracy of the CSC measurement is lower than that of the WL measurement for the first several hundred epochs. In other words, certain time smoothing is needed for CSC measurements to reaching a relatively high accuracy. In addition, CSC measurements can be regarded as a phase measurement with ambiguity without fixing to integer, but roughly calculated by the following expression:

(25) $$\frac{1}{n}\sum_{i=1}^{n}\left(P_{\left(i\right)}-\phi_{\left(i\right)}\right)$$

Therefore, the accuracy of CSC measurement also depends on whether the hardware delays of the signals are relatively stable and how long the multipath errors and observational noise can be eliminated or largely reduced. According to [41], the stability of BeiDou between-receiver differential code biases shows a dependence on satellite type, which has impacts on the carrier-smoothing performance.

In the medium baselines case, atmospheric errors also have an impact on positioning, because the atmosphere delays are not considered when implementing positioning for both algorithms. For tropospheric delays, the impacts is identical for both methods. For ionospheric delays, however, the impacts are different, because they dependent on frequency. The ionospheric scale factors of WL and CSC measurements are as follows:

(26) $$\{\begin{array}{l} \beta_{\phi_{WL}}=\frac{f_{1}^{2}}{f_{1}-f_{2}}\left(\frac{1}{f_{1}}-\frac{1}{f_{2}}\right)=-1.293\\ \beta_{\bar{P}}=1 \end{array}$$

As can be noticed, the ionospheric scale factor of WL measurements is a little higher than that of CSC measurements. Taking atmospheric errors into account, the user range error (URE) of WL and CSC measurements can be computed as follows:

(27) $$URE_{WL}=\sqrt{\sigma_{\phi_{WL}}^{2}+\sigma_{trop}^{2}+\beta_{WL}^{2}\cdot \sigma_{iono}^{2}}$$

(28) $$URE_{\bar{P}}=\sqrt{\sigma_{\bar{P}}^{2}+\sigma_{trop}^{2}+\sigma_{iono}^{2}}$$

Table 4 gives two sets of error budgets to roughly represent the short and medium distance cases. The errors refer to original measurements on the first frequency. Based on these error budgets, $URE_{WL}$ and $URE_{\bar{P}}$ for both short baselines and medium baselines are computed, as shown in Table 5. For $URE_{\bar{P}}$, we assume that the measurements have been implemented enough time carrier smoothing. The results indicate that the positioning accuracy of both methods will be mainly affected by the atmospheric errors in medium baselines. Therefore, the positioning accuracy will be approximately in the decimeter level.

**Table 4:** DD phase error budgets for short and medium distance [26].

Error Factors	Noise	Troposphere	Ionosphere
Short distance (0–10 km)	≈0.6 cm	<1 cm	<10 cm
Medium distance (10–100 km)	≈0.6 cm	<5 cm	<40 cm

**Table 5:** URE of CSC and WL measurements.

Case	$URE_{\bar{P}}$	$URE_{WL}$
Short distance (0–10 km)	0.107 m	0.136 m
Medium distance (10–100 km)	0.405 m	0.520 m

## 5. Data Collection and Processing Strategies

Observations of one short baseline and six medium baselines collected in static mode are used for our experiments. The baseline information is presented in Table 6. CUTA-CUT2 is located at the Curtin University of Technology (CUT, Perth, Australia). The data were collected on July 20 (DOY 201), 2015. The stations of the six medium baselines are located in Wuhan (China). The data were collected on February 22 (DOY 053), 2014. All of the data were obtained using Trimble NetR9 receivers, which can receive both GPS and BDS signals. The sampling rate of is 30 s for CUTA-CUT2 and 1 s for the six medium baselines.

**Table 6:** Baseline information.

Baseline	Distance
CUTA-CUT2	8.4 m
WHCD-WHHP	47.2 km
WHHN-WHHP	68.8 km
WHEZ-WHCD	83.9 km
WHEZ-WHHN	77.0 km
WHEZ-WHHP	74.5 km
WHHN-WHCD	28.7 km

For the DFWL-DGNSS method, observations on B1 and B2 frequencies are used for the test. For ORC-DGNSS method, we use the code measurement on B1 frequency for positioning. As to CSC-DGNSS, code measurements on one frequency and carrier phase measurement on two frequencies have to be used for carrier-smoothing. For GPS, we use code measurement on L1 and carrier phase measurements on L1 and L2. For BDS, we use code measurement on B1 and carrier phase measurements on B1 and B2. Details of the data processing strategy are summarized in Table 7.

**Table 7:** Processing strategy.

Options	Processing Strategy
Ephemeris	BeiDou broadcast ephemeris
Elevation cutoff angle	15°
Observation weighting	Elevation-dependent weightingRatio between standard deviation of phase and standard deviation of code: 1:100

## 6. Empirical Analysis

In this section, the fixing rate and positioning accuracy are evaluated empirically with observational data.

### 6.1. Empirical Fixing Rate

Using observations of the six medium baselines presented in Table 6, the biases of the float ambiguities for the WL, EWL-I and EW-II combinations relative to their corresponding true integer ambiguities are computed. The float ambiguities of the WL combination are computed based on the HMW combination. The float ambiguities of the EWL-I and EWL-II combinations are computed based on Equations (3) and (5), respectively.

**Figure 2:**
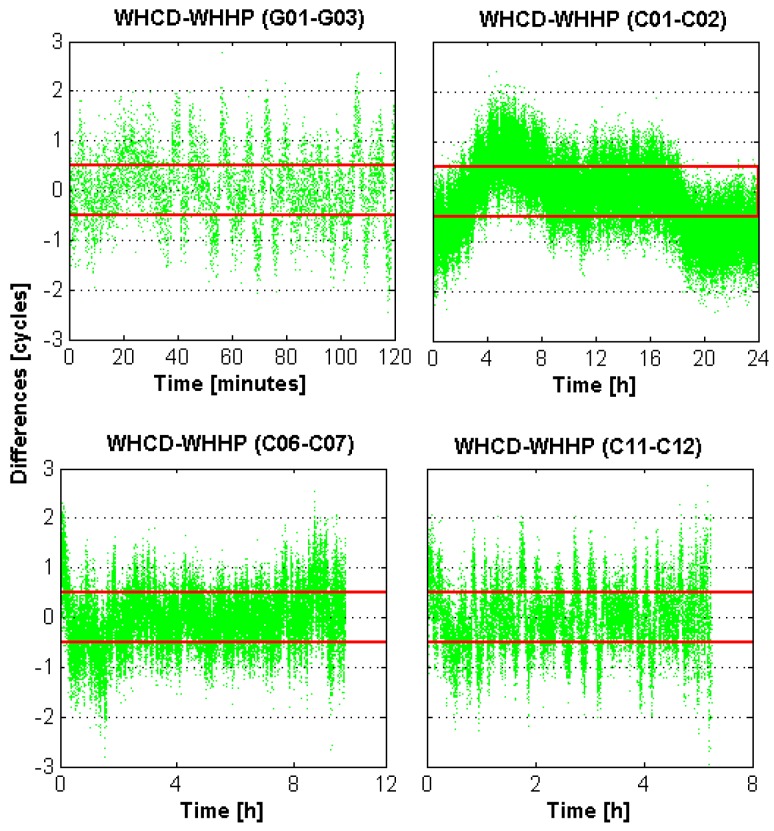
Dual-frequency WL float ambiguities of the GPS and BDS satellites for baseline WHCD-WHHP. The red line indicates the threshold for rounding. Green dots indicate the values of WL float ambiguities.

Figure 2 shows the biases of the WL float ambiguities of WHCD-WHHP in DOY-053 of 2014 for different types of satellites as examples. It can be noticed that the WL float ambiguities exhibit large errors with respect to the WL integer ambiguities. The maximum error is close to three cycles. Obvious systematic errors can be observed from the WL float ambiguities of the BDS GEO satellites (C01-C02), which are mainly caused by the systematic multipath errors of GEO satellites [42,43].

**Figure 3:**
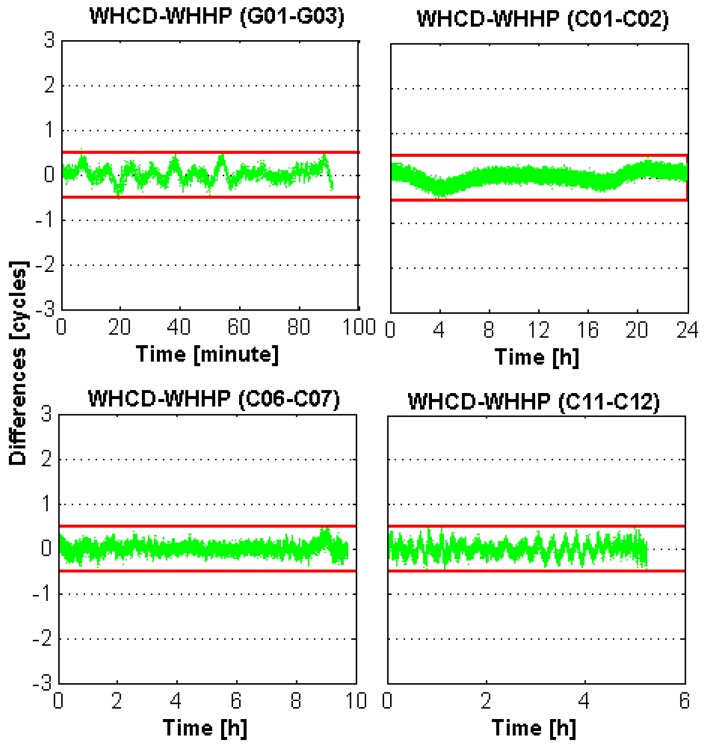
EWL-I time series of the GPS and BDS satellites for baseline WHCD-WHHP. The red line indicates the threshold of rounding. Green dots indicate the values of EWL-I combination.

**Figure 4:**
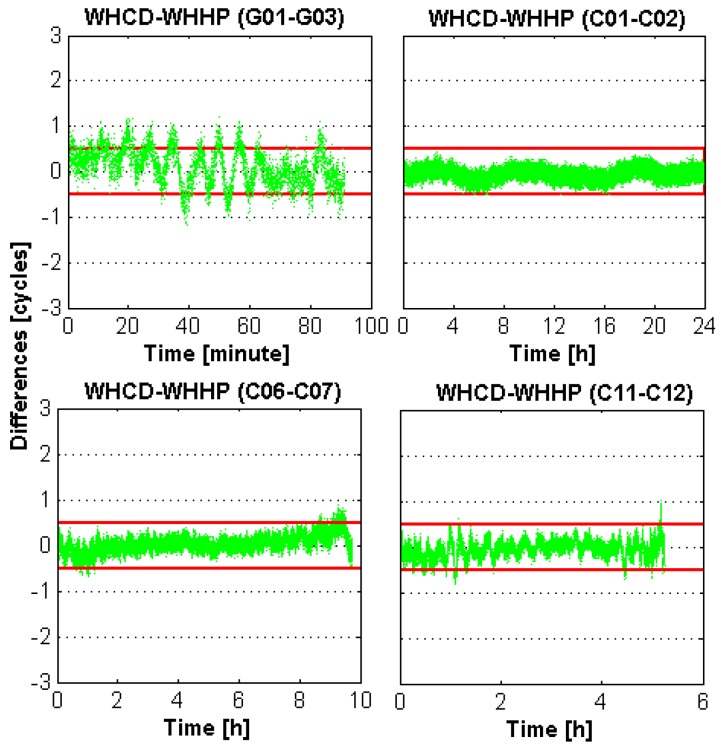
EWL-II time series of the GPS and BDS satellites for baseline WHCD-WHHP. The red line indicates the threshold of rounding. Green dots indicate the values of EWL-II combination.

Figure 3 and Figure 4 present the biases of the EWL-I and EWL-II float ambiguities of WHCD-WHHP in DOY-053 of 2014 for different types of satellites. It can be noticed that the errors of the EWL-I and EWL-II float ambiguities with respect to their integer ambiguities is smaller than 0.5 cycles most of the time, which is much smaller than those of the WL float ambiguities. The results of the EWL-II combination are a little lower than that of the EWL-I combination, which is mainly because the atmospheric errors are not fully eliminated for the EWL-II combination model. Furthermore, the fixing rate of GPS results is a little lower than that of BDS. This may be caused by the larger multipath errors of GPS code measurements. Fortunately, the fixing rate is extremely high for both the EWL-I and EWL-II combinations.

We also computed the AR fixing rate of WL, EWL-I and EWL-II using observations of the six medium baselines, as shown in Table 8. The fixing rate of the WL combination is computed based on the HMW combination. The fixing rates of the EWL-I and EWL-II combinations are computed based on Equations (3) and (5), respectively. As can be noticed from this table, the fixing rate of both BDS EWL-I AR and EWL-II AR is close to 100%, though the fixing rate of EWL-II AR is a little lower. The fixing rate of WL AR using the HMW combination, however, is lower than 65%. Specifically, the fixing rate of GPS WL AR is even lower than 60%. These results indicated that we can obtain WL measurements using single-epoch triple-frequency signals with extremely high success rate, which is impossible in the dual-frequency case.

**Table 8:** Fixing rate of GPS/BDS AR. The fixing rate of the WL combination is computed based on the HMW combination. The fixing rates of theb EWL-I and EWL-II combinations are computed based on Equations (3) and (5), respectively.

Combinations	GPS MEO	BDS GEO	BDS IGSO	BDS MEO
WL	59.30%	62.45%	65.11%	63.31%
EWL-I	99.90%	100.00%	99.98%	99.99%
EWL-II	82.18%	99.98%	98.57%	99.35%

**Figure 5:**
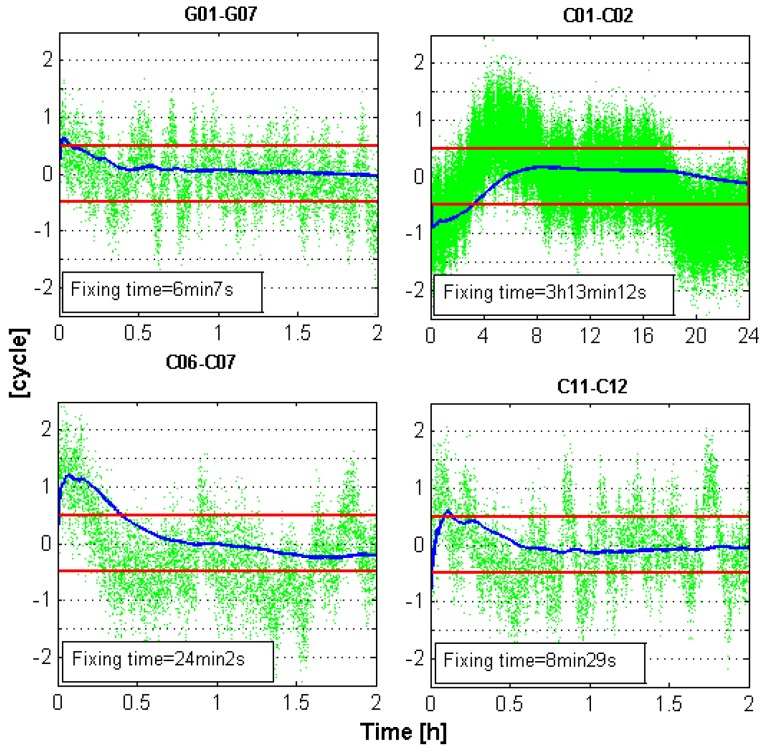
Averaged values of the HMW combination for baseline WHCD-WHHP. The red lines indicate the threshold of rounding. Green dots indicate the single-epoch values of the HMW combination. Blue lines indicate the averaged value of the HMW combination.

Multi-epoch smoothing or averaging algorithms also can be used to obtain the right solution. In this case, the smoothing time is very important. We compared the time needed for smoothing for different combinations. The time needed for achieving 100% fixing rate for dual-frequency signals and triple-frequency signals are computed. Figure 5 presents the averaged double-differenced HMW series of baseline WHCD-WHHP *versus* e time as an example. As can be noticed, several minutes to several tens of minutes are needed to obtain the correct wide-lane integer ambiguities for the BDS IGSO and MEO satellites. The time needed for GEO satellites is even longer. The minimum time for reaching 100% fixing rate is listed in Table 9. As can be noticed from this table, the needed time for obtaining the right solution for GPS dual-frequency signals and BDS dual-frequency signals are tens of minutes and several hours, respectively, which are much longer than that of GPS triple-frequency signals and BDS triple-frequency signals. These results indicate that implementing WL AR in a short time span using dual-frequency signals cannot be achieved. This is rather severe for BDS GEO satellites, since its multipath impact is large [42,43]. However, when triple-frequency signals are provided, the impact of noise and multipath of code measurements can be largely reduced by forming EWL combinations. WL measurements can thus be obtained with high fixing rates by linear derivation, which can be used for positioning.

**Table 9:** Minimum time needed for reaching 100% fixing rate. The results of the WL combination are computed based on the HMW combination. The results of the EWL-I and EWL-II combinations are computed based on Equations (3) and (5), respectively.

Combinations	BDS GEO	BDS IGSO	BDS MEO
WL	24 h	1 h	45 min
EWL-I	1 s	3 s	2 s
EWL-II	2 s	25 s	25 s

### 6.2. Empirical Accuracy

**Figure 6:**
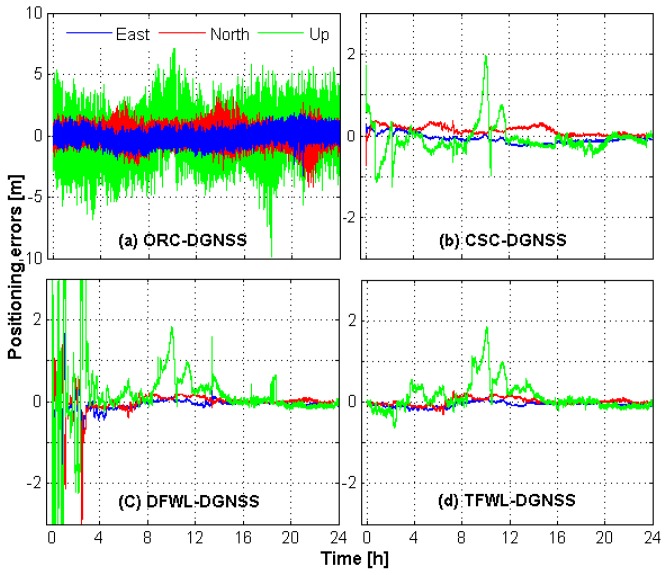
Positioning errors of ORC-DGNSS, CSC-DGNSS, DFWL-DGNSS and TFWL-DGNSS for baseline WHCD-WHHP.

Observational data of seven baselines listed in Table 6 are used for this test. The rounding threshold of the DFWL-DGNSS method is set as 0.2 cycles to guarantee reliable results. The ILS algorithm is applied for AR for TFWL-DGNSS method. Ratio test [44] is used to validate the ambiguity results. The threshold is set as 3.0 for the ambiguity validation of both EWL-I and EWL-II. Figure 6 shows the positioning errors of ORC-DGNSS, CSC-DGNSS, DFWL-DGNSS and TFWL-DGNSS obtained from BDS observations for baseline WHCD-WHHP as an example. As can be noticed, the positioning accuracy of ORC-DGNSS is much lower than those of the other three methods. The positioning accuracy of DFWL-DGNSS and TFWL-DGNSS are almost comparable to that of CSC-DGNSS. Theoretically, the accuracy of DFWL-DGNSS and TFWL-DGNSS will be equivalent each other once ambiguities are fixed. However, the errors of some epochs for DFWL-DGNSS are larger than that of TFWL-DGNSS, especially during the first several hours. This can be attributed to two reasons. Firstly, in some epochs, many WL observations are excluded because the fractional parts of their float ambiguities exceed the rounding threshold, thus the observations which can be used for positioning is few. The second reason is that there are some wrongly fixed wide-lane ambiguities in some epochs. We also can see that the positioning errors of CSC-DGNSS in the first several tens of epochs are relatively large. This is because the noises of the code measurements have not yet been smoothed to a rather low level.

**Figure 7:**
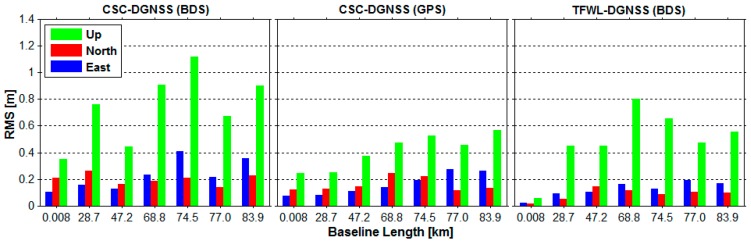
RMS of the positioning errors *versus* the baseline length. The left panel is for CSC-DGNSS results derived from BDS observations, the middle panel is for CSC-DGNSS results derived from GPS observations whereas the right panel is for TFWL-DGNSS results derived from BDS observations.

Figure 7 shows the RMS of the positioning errors for the seven baselines. In order to figure out whether the positioning accuracy is related to the baseline length, the results are ordered by the baseline distance. For comparison, the CSC-DGNSS results obtained from GPS observations are also presented. It is shown that the positioning accuracy is somehow dependent on the baseline distance. For short baselines, the positioning accuracy is relatively high, especially for TFWL-DGNSS results. For medium baselines, the accuracy of most results is lower than that of the short baseline case. As the baseline length increases, the positioning accuracy shows a decreasing trend. Some abnormal cases are seen for BDS results, which may be caused by the systematic multipath errors of BDS code measurements [43]. Fortunately, the positioning accuracy of TFWL-DGNSS for all the six medium baselines is at the decimeter level.

Table 10 presents the averaged RMS of the positioning results of ORC-DGNSS, CSC-DGNSS and TFWL-DGNSS for the six medium baselines. As can be seen, the accuracy of ORC-DGNSS is 5–7 dm in horizontal and lower than 18 dm in the up direction, much lower than those of the CSC-DGNSS and TFWL-DGNSS results. The accuracy of TFWL-DGNSS is 1.0–1.5 dm in horizontal and about 6 dm in the up direction. The accuracy of CSC-DGNSS for BDS observations and GPS observations is also different. The accuracy of CSC-DGNSS with BDS observations is 1.5–2.5 dm in horizontal and about 8 dm in the up direction, which is lower than that of TFWL-DGNSS in both the horizontal and up directions. The accuracy of CSC-DGNSS with GPS observations is about 1.7 dm in horizontal and about 4.5 dm in the up direction, which is a little lower than that of TFWL-DGNSS in the horizontal, but a little higher than that of TFWL-DGNSS in the up direction.

**Table 10:** Averaged RMS of CSC-DGNSS and TFWL-DGNSS. Units are in meters.

Method	East	North	Up	3D
ORC-DGNSS (BDS)	0.528	0.642	1.713	1.904
CSC-DGNSS (BDS)	0.248	0.187	0.790	0.852
CSC-DGNSS (GPS)	0.176	0.164	0.441	0.507
TFWL-DGNSS (BDS)	0.140	0.099	0.565	0.593

**Figure 8:**
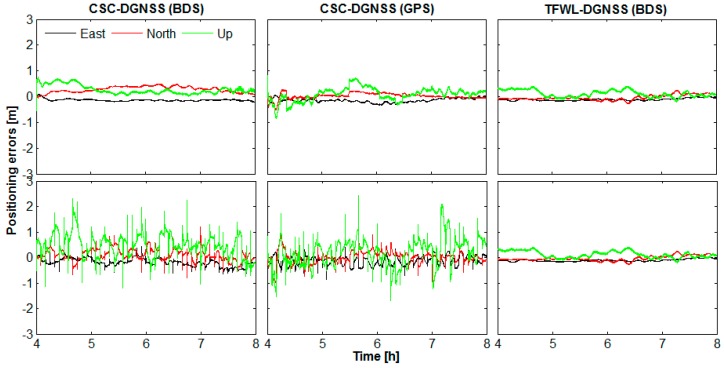
Positioning errors of the CSC-DGNSS and TFWL-DGNSS in both the signal interruption case and no signal interruption case for baseline WHCD-WHHP. The top panels are for the results without simulated signal interruption, whereas the bottom panels are for the results with simulated signal interruption.

In order to evaluate the impact of the signal interruption to these methods, we processed 4 h of WHCD-WHHP data with simulated signal interruptions. The simulation is implemented by reinitializing all the estimating parameters for every 300 s during the processing. The results with no signal interruptions are also obtained for comparison. Figure 8 shows the positioning errors of CSC-DGNSS and TFWL-DGNSS in both the signal interruption case and no signal interruption case for baseline WHCD-WHHP. As can be noticed, the CSC-DGNSS results of both BDS and GPS are affected by signal interruptions. Relatively high accuracy can only be reliably achieved when certain time smoothing is implemented after signal interruption for CSC-DGNSS. TFWL-DGNSS, however, is almost not affected by signal interruption. Since single-epoch AR can be implemented with very high success rate, high accuracy positioning results can be obtained immediately after signal interruption.

**Figure 9:**
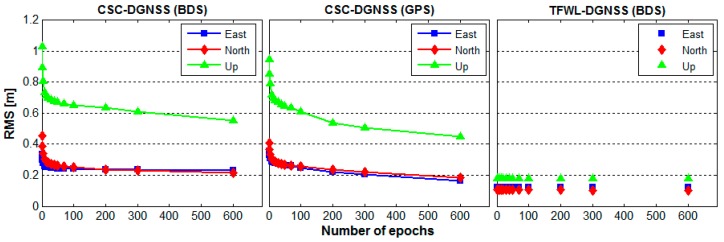
Positioning accuracy *versus* time for CSC-DGNSS and TFWL-DGNSS. The left panel is for CSC-DGNSS results derived from BDS observations, the middle panel is for CSC-DGNSS results derived from GPS observations whereas the right panel is for TFWL-DGNSS results derived from BDS observations.

By splitting the observational data into different time spans, ranging from several seconds to several minutes, we also computed the accuracy *versus* the time of CSC-DGNSS and TFWL-DGNSS. The results are presented in Figure 9. As can be seen, for CSC-DGNSS, the shorter the time span, the lower the accuracy is. Relatively high accuracy only can be achieved after 60–200 s of data are used. In contrary, the accuracy of TFWL-DGNSS remains stable regardless of the time span. These results display the advantage of TFWL-DGNSS for obtaining high accuracy positioning results rapidly.

## 7. Conclusions

In this paper we present a wide-lane positioning algorithm based on BDS triple-frequency signals, which can implement instantaneous real-time kinematic positioning with decimeter level positioning accuracy over medium distances. The fixing rate and positioning accuracy are carefully analyzed theoretically first, and then verified empirically by processing the data sets of seven baselines. The positioning performance is compared with that of carrier-smoothed code positioning.

The results indicates that the AR fixing rate of triple-frequency WL method with BDS observations can reach over 98%, which is much higher than that of DFWL-DGNSS. Decimeter level accuracy can be achieved by TFWL-DGNSS method, which is comparable to that of CSC-DGNSS method when using continuous observations. Signal interruption simulation experiments indicate that relatively high accuracy positioning results cannot be immediately obtained with the CSC-DGNSS method when signal interruption happens. However, they can be achieved using the TFWL-DGNSS method. The results imply that a high AR fixing rate and relatively high positioning accuracy can be achieved for the TFWL-DGNSS method with single-epoch or very short time observations, representing a significant advantage compared to the CSC-DGNSS method. The presented method can be an alternative method for the users requiring decimeter level rapid positioning results over medium distances. In future studies, the atmospheric errors should be considered to further improve the positioning accuracy. In addition, triple-frequency observations of the other GNSS systems can be added to improve the performance.
